# Do anaesthesiologists in pre-hospital care need concomitant clinical practice?

**DOI:** 10.1186/1757-7241-21-S1-S7

**Published:** 2013-05-28

**Authors:** SJM Sollid, M Sandberg, A Nakstad, P Bredmose

**Affiliations:** 1 Air Ambulance Dept., Oslo University Hospital, Oslo, Norway; 2 Norwegian Air Ambulance Foundation, Norway; 3 University of Stavanger, Stavanger, Norway; 4 University of Oslo, Oslo, Norway

## Background

Previous studies have documented that advanced life support (ALS) provided by anaesthesiologists in emergency medical service (EMS) leads to life years gained [1]. Especially interventions legally and formally related to anaesthesiology were judged as crucial: endotracheal intubation (ETI), chest tube insertion and anaesthesia induction. These interventions require extensive training and experience, and probably retraining and regular practice, to be performed safely. The need for retraining and practice is probably related to actual exposure to these interventions in clinical practice. To explore if there is a need for retraining and practice, we have investigated how often these interventions are performed by individual anaesthesiologists in a typical Norwegian physician manned EMS.

## Method

Advanced interventions performed by the anaesthesiologist on call at three helicopter and car EMS units and one search-and-rescue unit were reported on a standard form for 12 months during 2011. Data on advanced airway management and surgical- and anaesthesiological procedures are reported here. Based on the number of prehospital working hours reported by each physician we calculated the number of procedures performed per 24-hour for each individual. With the mean value for each procedure per 24 hours per physician we have calculated the expected number of procedures performed in one year based on full time HEMS physician engagement equivalent to 84 24-hour shifts. The Data Protection Official of all involved institutions approved the data collection as a quality improvement project.

## Results

We received forms from 82.7% of the 1460 duty-days covered by the study. Thirty-six individual physicians reported on their activities during the study and the mean number of duty hours per physician was 804.9 hours (IQR 273, 1315.5). The expected number of procedures performed per year per physician is listed in Table 1.

**Table 1:** Expected number of procedures performed by the individual HEMS physician in full time HEMS engagement per year and expected time between each procedure exposure.

Procedure	n/year	Time between exposure
Paediatric ETI	1.4	8.5 months
Adult ETI	24.8	2 weeks
Surgical airway	0.04	25 years
Chest tube insertion	1.7	7 months
Anaesthesia induction	19.6	2.5 weeks

## Conclusion

Anaesthesiologists in Norwegian EMS can expect relatively little exposure pre-hospital to crucial life-saving interventions. Concomitant in-hospital clinical practice and other skills retraining should therefore be maintained to ensure adequate proficiency in these interventions.
